# Editorial: Case reports in cardio-oncology: 2022

**DOI:** 10.3389/fcvm.2023.1235015

**Published:** 2023-07-13

**Authors:** Michael S. Ewer, Syed Wamique Yusuf, Reto Asmis, Jun-ichi Abe

**Affiliations:** ^1^ MD Anderson Department of Cardiology, Internal Medicine Division, The University of Texas, Houston, TX, United States; ^2^ Department of Internal Medicine, Wake Forest School of Medicine, Winston-Salem, NC, United States

**Keywords:** cardiooncology, cardiotoxicity after chemotherapy, history of cardio-oncology, adverse cardiac events, cardiac monitoring of cancer patients

**Editorial on the Research Topic** Case reports in cardio-oncology: 2022

One of the early references to an effect of cancer on the circulatory system was the observation by Armand Trousseau in the 1860s that vascular inflammation could be an early manifestation of the presence of a malignancy (1). Recurrent or migratory non-infectious vascular disease would become an important clinical sign that continues to draw the attention of clinicians to the possibility of occult malignancy. Cancer and cardiovascular disease are the two most common causes of mortality worldwide (2). Improvements in cancer therapy have led to the increasing number of cancer survivors. In the USA, an estimated 18 million individuals with a history of cancer were alive on January 1, 2022 (3). A sizeable number of these patients have cardiotoxicity related to radiation and or chemotherapy. In addition, amongst older patients diagnosed with cancer, heart and vascular disease are the most frequent concomitant conditions (4). Hence, in clinical practice, it is not uncommon for a clinician to face a patient who has a concomitant diagnosis of both cancer and cardiovascular disease.

Cardiovascular disease attributable to malignancy and, in more recent times, to the treatment of malignancy, has expanded. Interventions to mitigate preventable acute and long-term cardiovascular sequelae of the disease or its treatment have evolved into a discipline now referred to as cardio-oncology.

The era of cardio-oncology emerged with the introduction of therapeutic agents, most notably in the late 1960s and early 1970s with the introduction and expansion of anthracyclines (5). Exposure to anthracyclines resulted in cardiac failure that was dose-dependent when given in sufficiently high dosages, resulted in severe cardiac failure and death (6). Several important characteristics emerged: first, the toxicity could be quantified by ultrastructural changes in the myocyte when studied by electron microscopy (7). Dosages could be individualized, as not all exposed individuals showed equal sensitivity to the cardiotoxic effects, and some could tolerate doses several times greater than others. Additionally, risk factors for augmented sensitivity to these agents were explored and identified. Initially, entities such as hypertension and valvular lesions were noted to be risk factors, but the more modern view emerged that any entity that had either damaged the heart or that made it more susceptible to ongoing damage was a risk factor. Throughout the 1980s cardiac biopsies were undertaken to assess toxicity and were graded according to the toxicity scale initially proposed by Billingham and later modified by Mackay that allowed dosages to be individualized (8).

As non-invasive techniques improved, cardiac ejection fraction became the parameter of choice for cardiac surveillance, and both cardiac ultrasound and nuclear imaging in the form of multi-gated (MUGA) scans were widely used. However, it was recognized that while the cardiac biopsy showed and clearly quantified the injury to the myocyte, the ejection fraction could only detect the effects of the injury when compensatory reserves were sufficiently impaired. By the early 1980s, it was evident that ejection fraction reduction was a late consequence of anthracycline injury and was clearly not an optimal parameter for the guidance of anthracycline therapy (9). At that time sufficient interest had accumulated regarding risk assessment of cancer patients that the first mention of a cardio-oncologist (“oncologic cardiologist”) appeared in a major journal (9). Later, the fact that myocyte apoptosis-released troponin helped to solidify the concept that the anthracycline injury occurred early and could be quantified at the time of injury despite the fact that the impact of the injury might only come to the forefront years or even decades later (10).

As different therapeutic strategies entered the oncologic armamentarium other adverse events became recognized. Coronary spasm associated with 5-fluorouracil, while recognized, did not reach the threshold of requiring pre-evaluation or on-going surveillance. The approach to other modalities including radiation was largely reactive at that time; if a patient experienced an event or a hypertensive crisis, referral to a cardiologist might ensue to optimize management. By the 1990s, the taxanes had entered standard chemotherapy regimens, but cardiovascular sequelae were not a major concern; the associated bradycardia was generally transient and did not require the expertise of a cardiologist. Cyclophosphamide caused hemorrhagic myocarditis, but this was usually seen when high dosages were administered; high-dose usage and hemorrhagic myocarditis, while recognized, was uncommon, and the cardiologist's role was generally one of providing supportive care.

With the development of the monoclonal antibody trastuzumab, cardiac concerns regarding that agent, and later other novel agents, came to the forefront. An early report noted that, in patients with metastatic disease treated with an anthracycline, cyclophosphamide, and trastuzumab, class III or IV cardiac failure occurred in 27 percent (11). As reports of cardiac events following administration of trastuzumab appeared, there was great concern regarding the observed toxicity. It was speculated that it could be direct toxicity, but also might be the result of a synergistic effect, or a surveillance artifact (12). At that time, most thought that the reported toxicity was mechanistically similar to that seen with the anthracyclines and, therefore, would suggest that great caution was needed when using this highly effective medicine. Concerns were raised, especially in view of the intended role of trastuzumab in the adjuvant breast cancer setting, where many women could be exposed to this agent who, in fact, did not have residual cancer.

In 2005, it became clear that the cardiotoxicity of trastuzumab was fundamentally different from that of the anthracyclines and the concept of a new form of cardiotoxicity, now often referred to as Type II, was introduced (13). Trastuzumab did not show the ultrastructural changes characteristic of the anthracyclines and the decreased cardiac contractility was often transient. While some found the categorization of Type II agents, i.e., agents that do not directly cause myocyte injury as do anthracyclines, to be superficial or overly simplistic, the categorization had two huge effects: first, it became universally recognized that some forms of cardiac dysfunction following cancer treatment behaved differently from that seen with the anthracyclines. Perhaps more importantly, it allowed some of the major clinical trials to continue despite the recognition of decreasing ejection fractions; the results of those trials ultimately led to the approval of trastuzumab in the adjuvant setting, a huge breakthrough that resulted in a major life-sparing innovation in the treatment of HER2 positive breast cancer.

Questions regarding the cardiotoxicity of trastuzumab, and later of other monoclonal antibodies and tyrosine-kinase inhibitors, continued to raise concerns. The ability to mine expanding databases further added to the confusion regarding the true clinical risk of these newer agents as well as to questions about the ideal way to screen patients prior to their receiving anti-cancer treatment as well as what surveillance during and following treatment should be undertaken. As more studies were entered into the literature, the idea that some reports might have included false positive data and confounding factors, i.e., real changes in cardiac function related to factors other than a specific anti-cancer treatment might explain the disparity between the number of reported events and the clinical perception by many oncologists regarding the safety of some of these newer agents (14, 15). Concerns were sufficiently large that cardiac surveillance exploded and monitoring guidelines continue to suggest that surveillance was essential despite little evidence that extensive monitoring provided meaningful clinical benefit. In many instances guidelines were not followed (16). Additionally, both early and late sequalae of radiation were recognized and, while cardioprotective strategies to protect the heart from radiation exposure were developed, long-term late expression of radiation injury remains an important consideration in the surveillance of patients previously exposed to radiation (17).

A new era ensued with new concerns of cardiac damage as the vitally important and innovative group of anti-cancer agents, the immune checkpoint inhibitors, was introduced. These agents were associated with a rare but often lethal form of myocardial inflammation that continues to receive considerable attention. Risk factors are gradually emerging, but it is now clear that combinations of these agents have resulted in increased incidences of toxicity. Estimates of serious events suggest that clinically relevant myocardial inflammation occurs in about 1% of treated patients, but sub-clinical inflammation may occur in a greater proportion of patients. Treatment strategies usually include steroid administration, but other interventions are emerging (18).

As we move forward, the combined efforts of cardiologists and oncologists to optimize survival and quality of life of cancer patients will continue and will become increasingly important as remissions expand for increasingly long periods and cures become more common. The goal of cardio-oncology will be a more focused approach to help achieve an optimal balance between the risks of cancer treatment that impact the cardiovascular system and that include secondary burdens that affect quality of life with true short and long-term benefits of these interventions. Our ability to integrate new information and to apply new analytical techniques to achieve this balance will be one of the future goals of cardo-oncology, and one that is highly likely to continue to be important and potentially hugely meaningful.

As we explained above, cardio-oncology is a rapidly evolving field that focuses on the intersection between cardiovascular diseases and cancer. It has become increasingly important because advancements in cancer treatment have improved cancer survival rates, but they can also lead to cardiovascular complications. Therefore, it is crucial that cardio-oncologists understand and learn to mitigate the cardiovascular side effects of cancer therapies, optimize treatment strategies, and improve the overall outcomes and quality of life for cancer patients. To accomplish this goal, a multidisciplinary approach with collaboration between oncologists and cardiologists is essential. Both specialists bring their unique expertise, enabling comprehensive patient care. Working together, they can tailor cancer therapies to minimize cardiac risks and develop cardiovascular management strategies for cancer patients.

Cardio-oncology is a relatively new discipline, and this strengthens the importance of case reports. Such reports provide valuable insights into previously unknown or under-recognized cardiovascular complications of cancer treatments; many cardiotoxic sequalae, especially those that had not been anticipated from pharmacologic considerations or not seen in pre-clinical or early clinical studies, were first brought to our attention through case reports. These reports serve as the foundation for generating new knowledge, often through augmented surveillance and retrospective analysis. This, together with our understanding of the mechanisms, risk factors, and management strategies for such complications is crucial as we strive to place such events in perspective. Case reports also help in developing guidelines and best practice algorithms for cardio-oncology. We understand that there is a discussion to eliminate *Case Reports* because this section may negatively affect the impact factor of the journal. However, we would like to challenge this perception as we believe that Case Reports are critically important to the still young and evolving field of Cardio-Oncology and provide us with state-of-the art information. We are facing many new drugs and interventions. By focusing on cardio-oncology cases and obtaining more knowledge from each case, we can improve our understanding and ultimately improve patient outcomes, enhance survivorship, and optimize cancer treatment strategies for individuals with concurrent cancer and cardiovascular disease.
